# Socioeconomic Inequalities and Ethnic Discrimination in COVID-19 Outcomes: the Case of Mexico

**DOI:** 10.1007/s40615-023-01571-z

**Published:** 2023-04-11

**Authors:** Andrea Salas-Ortiz

**Affiliations:** Centre for Health Economics, University of York, Alcuin Block A, York, YO105DD North Yorkshire England, UK

**Keywords:** Health inequities, Discrimination, COVID-19, Indigenous people, Oaxaca decomposition, Mexico

## Abstract

In Mexico, Indigenous people were hospitalised and killed by COVID-19 at a disproportionate rate compared to the non-Indigenous population. The main factors contributing to this were poor health conditions and impoverished social and economic circumstances within the country. The objective of this study is to examine the extent to which ethnic disparities are attributable to processes of structural discrimination and further explore the factors that exacerbate or mitigate them. Using administrative public data on COVID-19 and Census information, this study uses the Oaxaca-Blinder decomposition method to examine the extent to which disparities are illegitimate and signal discrimination against Indigenous people. The results show that although ethnic disparities were mainly attributable to observable differences in individual and contextual characteristics, 22.8% (*p* < 0.001) of the ethnic gap in hospitalisations, 17.5% in early deaths and 16.4% in overall deaths remained unexplained and could potentially indicate systemic discrimination. These findings highlight that pre-existing and longstanding illegitimate disparities against Indigenous people jeopardise the capacity of multi-ethnic countries to achieve social justice in health.

**Supplementary Information:**

The online version contains supplementary material available at 10.1007/s40615-023-01571-z.

## Introduction

Epidemics have disproportionate effects on indigenous populations and tend to perpetuate pre-existing and longstanding social, economic and health inequities [1]. Notwithstanding, a syndemic state can take place when two or more epidemics occur simultaneously and socioeconomic inequalities exist within societies [2]. Mexico is a multi-ethnic country with 21.5% of its population self-identifying as Indigenous [3] all across the country.^1^ Before the COVID-19 epidemic hit Mexico (officially on February 27^th^ 2020) [4], this country was already facing a public health crisis driven by non-communicable diseases (NCDs). Additionally, economic inequality was high, with 20% of the poorest households earning 5% of the total income [5, 6].

Most of the time, epidemics have a bigger impact on Indigenous people than on the general population. Indeed, the outsized effects that Indigenous people faced during the pandemic were influenced by the impoverished social and economic characteristics they were facing when COVID-19 hit [7–11] such as limited access to health services, poor living conditions, low academic performance [12], elevated levels of poverty and low life expectancy [13–15]. The interaction between these pre-COVID-19 factors and vulnerable conditions contributed that Indigenous people obtained worse health outcomes during the pandemic. In this context, the objective of this study is to examine the extent to which these ethnic disparities are attributable to structural discrimination and further explore the factors that exacerbate or mitigate them. This analysis centres on examining the pre-COVID-19 vaccination period, covering a time span of 15 months to better understand how health shocks produce or deepen ethnic inequities.

Despite the boom in the quantitative literature about socioeconomic inequalities in the light of the COVID-19 pandemic, a relatively small number of researches have focused exclusively on the analysis of ethnic inequalities, [8–11, 15–17] and a few studies have analysed the particular case of Mexico [18, 19]. Undoubtedly, these studies have progressed in better understanding the link between COVID-19 and ethnic inequalities, yet little is known about ethnic health inequities. The central aim of this study is to advance on the ongoing literature about ethnic inequities and COVID-19 by distinguishing legitimate (justifiable) and illegitimate (unjustifiable) inequalities, the latter known as inequities in the distributive justice literature [20–22]. To follow this aim, this study capitalises on the features of the Oaxaca-Blinder (OB) decomposition method. This method assumes, upfront, that disparities across groups exist, in this case, an ethnic health gap. Thus, it focuses on breaking down this gap into an observed, or explained component, which captures justified (legitimate) inequalities, and an unobserved, or unexplained part, which captures unjustified (illegitimate) inequalities. On the one side, the observed part explains the outcome disparities by given differences in individual and contextual characteristics included as variables in the estimated models proposed by the method (age, sex, underlying conditions, household characteristics, etc.). On the other side, the unobserved part, explains disparities by given differences in the link between characteristics and outcomes, the estimated coefficients of the models. According to the OB approach, the observed part denotes justified inequalities: differences exist because individuals are different in their characteristics and circumstances, and this is acceptable. However, in the absence of justified explanations for the differences across groups through observable factors, the unobserved component reflects illegitimate disparities, and it is, therefore, a potential indicator of discrimination [23, 24].

In this study, discrimination is conceptualised as the exercise of informal or institutionalised practices “that deny equal treatment or produce unequal results for certain social groups, resulting in the deprivation or undermining access to rights and in the reproduction of social inequalities” [25, 26]. Discriminatory practices can be exerted not only by individuals but also by social and public entities. Indeed, some practices exerted by governmental bodies can deny or explicitly restrict access to different social and public spheres. There are practices that although do not deny any access to them, condition or limit the mobility within social spheres, e.g., give unjustified preference to some individuals over others [26]. Discrimination can be directly exerted, but it can also take an indirect or structural form that consists of confining access to basic rights and public services based on historical norms, routines, and social institutions [27]. Thus, illegitimate disparities and discrimination entail unjustified unequal treatment across individuals. The latter facilitates grasping the conceptual differences between inequalities and inequities. Inequality is about differences in the distribution of health across groups, whereas inequity refers to the extent to which this distribution is unfair, and therefore, implies a normative judgment [20].

A first discussion about the link between discrimination and health inequities between Indigenous and non-Indigenous people in Mexico is found in the work of Serván-Mori, Juárez-Ramírez, Meneses-Navarro, et al. (2022). This empirical study illustrated the identification of structural ethnic discrimination in effective coverage of maternal health care by using the OB decomposition method, [28] and emphasizing that discrimination is compounded by inequity: despite individuals having the same capabilities and characteristics, their membership in a social group conditions their access to and provision of health services. Thus, differences in access to basic rights and the provision of public services between social groups are illegitimate and could further contribute to reproducing inequities.

In this regard, this study offers relevant contributions to research by unveiling an unequal treatment between Indigenous and non-Indigenous people in Mexico during the first 15 months of the COVID-19 pandemic. Findings indicate that, although around 77–83% of the ethnic health gap is due to observable characteristics, a non-trivial part of the gap remains unexplained and could be signalling discrimination against Indigenous people in hospitalisations, early mortality, and mortality due to COVID-19. This shows profound discrepancies between high-level commitments to prioritise the welfare of Indigenous people and the social and health policies in place when the pandemic started.

This paper is structured as follows. The next section presents the study design, data sources, key variables, and the analytical strategy used. Next, the descriptive and decomposition results are described. The last section finalises with a discussion and conclusion around the main findings.

## Methods

### Study Design and Data Sources

A cross-sectional OB decomposition was conducted using as a main source of information the open administrative data on COVID-19, from the General Directorate of Epidemiology (Dirección General de Epidemiología (DGE)). Data from the General Directorate of Health Information (DGIS, in Spanish), the National Council on Population (CONAPO, in Spanish) and the National Institute of Statistics and Geography (INEGI, in Spanish) were also used.

#### Individual-Level Data

Data on COVID-19 was compiled in the National Epidemiological Surveillance System (SINAVE, in Spanish), a dataset administered by the DGE. This is an administrative and publicly available source of information that contains data about COVID-19 cases that seek medical attention in Mexico. For each case registered in the dataset, information such as place of residence, age, sex, nationality, ethnicity (whether a patient identifies as an Indigenous language speaker), migratory status, as well as the patient’s health institution affiliation and basic clinical information were collected. For an inpatient: admission date, symptom onset date, admission to ICU and/or date of death, polymerase chain reaction (PCR) test result (positive, negative, or pending) and for women tested, whether they were pregnant. Clinical information included the presence of underlying conditions such as pneumonia, chronic obstructive lung disease (COPD), asthma, immunosuppression, diabetes, obesity, hypertension, chronic renal and cardiovascular disease, other comorbidities and whether the patient was a smoker. In the dataset, these are all indicator variables, and no further information was provided. This publicly available dataset was updated every day since January first, 2020, and some of the variables had reporting delays [29]. For this analysis, the version released on the fourth of April 2021 is used.

The Mexican government did not follow a universal COVID-19 testing policy, therefore, only those with symptoms were eligible for a test. The data collection process (who, where, when and how) is described in detail in Supplementary Material A and Fig. A2. The results about testing, hospitalisation, and patient follow-up (discharge, or worsening condition where patients were admitted to ICU (intensive care unit) or passing away) were directly uploaded by the diagnostic facility or hospital according to test results. Since there was no variable available to identify a patient, cases with the same information about demographics and clinical history were matched and eliminated as duplicate observations.^2^ Patients with incomplete (pending results) or missing information about testing results and ethnicity were also excluded, as well as non-Mexican patients.

#### Contextual Data

For contextual data aggregated information at the municipality level was used, and the health infrastructure indicators came from the DGIS. Data on municipal socioeconomic marginalisation came from the CONAPO and information about the population dispersion from the INEGI. These municipal-level data were merged with the individual-level information to construct a cross-sectional dataset.

#### Study Participants

The sample for this analysis consisted of individuals born in Mexico that were hospitalised due to COVID-19 or died in a hospital because of COVID-19 complications between January 2020 and March 2021. This sample yields to 4,829,071 individuals, of which 4,797,799 are non-indigenous and 31,272 are indigenous people. Due to the inclusion of covariates and missing values in some variables, the sample size for the decomposition analysis is between 4,688,278 and 4,575,481 depending on the outcome.

### Main Variables

#### Ethnic Groups

Ethnicity is defined according to cultural criteria such as the language individuals mostly speak in their daily life. There is no official language in Mexico, but Spanish is mostly used by the government and spoken by most of the population. Along with Spanish, 68 Indigenous languages are also spoken across the country. Thus, for this study, the operational definition of ethnicity is a binary classification with two groups identified according to whether individuals speak an Indigenous language as their mother tongue or not. The group variable takes the value of one if an individual reported speaking an Indigenous language and zero otherwise.

#### COVID-19 Health Outcomes

This analysis focuses on hospitalisations and deaths due to COVID-19, and based on a past study, early mortality is also studied [19].

Specifically, the health outcomes analysed are:

- To be hospitalised due to COVID-19
- To die within 5 days of being hospitalised because of COVID-19-related complications (early dead) [19, 31].
- To die due to COVID-19-related complications, at any point in time.

These are binary variables and take the value of one if the event is true and zero otherwise.

These outcomes are meant to be interpreted as “bad” or “ill-health” that aim to reflect a worsening health condition related to people contracting COVID-19. Given the dataset used, this analysis is restricted to individuals that were only hospitalised or that died in health facilities [32, 33]. The study of these outcomes is appropriate for the study of potential structural discrimination since, in a context of a fragmented health system,^3^ they might indirectly reflect heterogeneity in the structural conditions and quality of care received by those infected by COVID-19.

#### Individual and Contextual Variables

Explanatory variables are divided into two categories, individual-level characteristics, and contextual circumstances. Individual-level data comprises demographics (sex and age), underlying health conditions (pneumonia, hypertension, diabetes, COPD, asthma, immunosuppression diseases, renal diseases, cardiovascular diseases, and others), risky health behaviours (obesity and being a smoker) and medical attention (testing waiting-time and health provider where the patient received medical attention). The institution where individuals received medical attention is captured by a variable indicating the type of institution where individuals were hospitalised or died. The six public health provider institutions were divided into social security institutions (IMSS, ISSSTE, PEMEX, SEDENA, and SEMAR) and health secretariat institutions (Federal and State Ministry of Health-owned hospitals). The inclusion of these variables is based on previous evidence about COVID-19 treatment differences across public health institutions [36, 37].

Contextual circumstances were included to account for conditions that individuals are exposed to and cannot change in the short run. For example, health infrastructure, levels of socioeconomic marginalisation and population dispersion. For these variables, data at the municipality-of-residence level are used, since it is an indirect but reliable way to proxy the social and economic deficiencies that can be correlated with health outcomes, and it is the most granular disaggregated data that can be obtained.

Specifically, the indicators established by the Mexican Ministry of Health for COVID-19 care were included in the analysis, these are the number of medical offices and hospital beds per 10,000 inhabitants. Socioeconomic marginalisation was proxied by an index summarising a range of welfare indicators related to education, housing, distribution of the population and monetary income. This index is constructed by the CONAPO using data from the 2020 National census. Since population dispersion is one of the most challenging aspects when providing public services in Mexico [38, 39], it was relevant to account for in this study. This was proxied by the percentage of urban localities within each municipality.

A further description of all these variables is found in Table A1.

### Analytical Approach

#### Descriptive Analysis

To understand the population’s profile and differences across groups, a descriptive overview of the data is first undertaken. Average inter-group differences in the covariates used by groups are calculated. Then, the mean differences in all outcomes are assessed to further justify that an ethnic gap decomposition is feasible. When variables are binary, these are expressed as proportions and the mean differences are also statistically tested.

#### Decomposition Analysis

To formally investigate the illegitimate COVID-19 outcome differences between Indigenous and non-Indigenous people, the aggregate and the detailed versions of the OB decomposition model are applied. Both are formally explained next.

#### Aggregate OB Decomposition

The OB method is based on a regression model where a health outcome is regressed against a set of covariates, which in this analysis are an individual’s health conditions and contextual circumstances, as previously explained. With no loss of generality, this is formally represented in the following structural function^4^:

1 $${Y}_{i}^{g}={m}^{g}\left({X}_{i},{\epsilon }_{i}\right)={\beta }_{0}^{g}+{\beta }_{1}^{g}{X}_{1i}+\dots +{\beta }_{k}^{g}{X}_{ki}+{\epsilon }_{i}^{g}$$

With $g=\mathrm{0,1}$ and ${Y}^{g}$ representing the health outcome for group g. ${X}_{k}$ depicts distinct factors that influence the outcome *Y*. *i* indexes individuals, *g* represents the comparison and reference groups and, ${\epsilon }_{i}^{g}$ is the idiosyncratic error term of the model. The model assumes *additive linearity*. This is: $m\left(X,\epsilon \right)=X{\beta }^{g}+{\epsilon }^{g},$ which implies that the effect of observed and unobserved characteristics is additively separable in *m(.)*. The model also assumes *zero conditional mean independence*: $E\left(\epsilon \right|X,G)=0$. Thus, the average group difference can be expressed as:

2 $$\begin{array}{l}{\Delta }^{\mu }=\mu \left({\mathrm{F}}_{Y|G=0}\right)-\upmu \left({\mathrm{F}}_{Y|G=1}\right)=E\left({Y}^{0}|G=0\right)-E\left({Y}^{1}|G=1\right)\\ \;\;\;\;\;\;=E\left({X\beta }^{0}+\epsilon |G=0\right)-E\left({X\beta }^{1}+\epsilon |G=1\right)\\ \;\;\;\;\;\begin{array}{l}=(E\left({X\beta }^{0}|G=0\right)+E(\epsilon |G=0))-(E\left({X\beta }^{1}|G=1\right)+E(\epsilon |G=1))\\ =E\left({X\beta }^{0}|G=0\right)-E\left({X\beta }^{1}|G=1\right)\end{array}\end{array}$$

Thus,

3 $${\Delta }^{\mu }=E\left(X|G=0\right){\beta }^{0}-E\left(X|G=1\right){\beta }^{1}$$

The decomposition of the average inter-group difference is based on the use of a counterfactual that depicts what would happen if the characteristics of one group were interchanged with the coefficients of the other group. This counterfactual is ${F}_{Y}^{0}|G=1$ and depicts the average expected outcome for group 1 if they had the characteristics of group 0. This is:

4 $$\begin{array}{c}\mu \left({F}_{{Y}^{0}}|G=1\right)=E\left(X{\beta }^{0}+\epsilon |G=1\right)\\ =E\left(X{\beta }^{0}|G=1\right)\end{array}$$

By subtracting and adding the counterfactual $E\left(X{\beta }^{0}|G=1\right)$ in Eq. (3), the *explained* and *unexplained* components are obtained, as follows:

5 $$\begin{array}{l}{\Delta }^{\mu }=E\left(X|G=0\right){\upbeta }^{0}-E\left(X|G=1\right){\beta }^{1}\\ \;\;\;\;\;\; =E\left(X|G=0\right){\upbeta }^{0}-E\left(X|G=1\right){\beta }^{0}+E\left(X|G=1\right){\upbeta }^{0}-E\left(X|G=1\right){\beta }^{1}\\ \;\;\;\;\;\;=\left(E\left(X|G=0\right)-E\left(X|G=1\right)\right){\beta }^{0}+E\left(X|G=1\right)({\beta }^{0}-{\beta }^{1})\\{\Delta }^{\mu }={\Delta }_{X}^{\mu }+{\Delta }_{\beta }^{\mu }\end{array}$$

${\beta }^{g}$ can be estimated using linear regression models, for example, ordinary least squares (OLS) models on the $G=g$ sub-sample and $E\left(X|G=g\right)$ is the vector of means of *X* in the same sub-sample.

Given that our dependent variables are binary, we use non-linear models. Under these models, $\mathrm{E}\left(\mathrm{Y}|\mathrm{X}\right)\ne \mathrm{F}(\overline{X }\upbeta )$. To solve this issue, we follow an extension of the OB decomposition using the logit function [41, 42], in which the decomposition of a nonlinear equation such as $Y=F\left(X;\widehat{\beta }\right)$ can also be written as:

6 $$\begin{array}{c}\widehat\Delta^\mu=\underbrace{\left[\frac1{N^0}\sum\nolimits_{G_i=0}F\left(X_i^0\widehat\beta^0\right)-\frac1{N^1}\sum\nolimits_{G_i=1}F\left(X_i^1\widehat\beta^0\right)\right]}_{Explained}+\\ \underbrace{\left[\frac1{N^1}\sum\nolimits_{G_i=1}F\left(X_i^1\widehat\beta^0\right)-\frac1{N^1}\sum\nolimits_{G_i=1}F\left(X_i^1\widehat\beta^1\right)\right]}_{Unexplained}\end{array}$$

Applied to the logit function,^5^${\widehat{\Delta }}^{\mu }$ denotes the predicted average difference in the coefficients of the binary outcome of interest and $F(.)$ represents the cumulative distribution function from the logistic distribution: $\frac{1}{1+{e}^{-X\beta }}$.

Equation (6) shows the two-fold and aggregate decomposition where the explained part focuses on differences in the variables included in the model holding constant the coefficients from group 0. The unexplained component takes group 1 as the reference group and focuses on differences in the regression coefficients from both groups.^6^ If this is modified, e.g., taking group 0 as reference, the results of the decomposition would change as well. This issue is known as the *indexing problem* and implies that results are not unique and depend on the group chosen as reference. The decision of which group to take as a reference should be made based on a preconception of the existence of discrimination. In this case, given the consistent evidence about the unequal and unfair social treatment that Indigenous people have in comparison to non-Indigenous in Mexico [12–15], it is believed that the assumption of discrimination against Indigenous people holds and therefore, the decompositions are undertaken using Indigenous people as the reference group.

#### Detailed OB Decomposition

For policy purposes, it is relevant to further identify and measure the main factors contributing to the explained and unexplained parts of the ethnic gap. This extension of the aggregate decomposition is known as the detailed OB decomposition and consists of subdividing each component and estimating the contribution of each explanatory variable ${k}^{th}$ [43]. However, the nonlinear model imposes the issue known as is the *path dependence problem* [43, 47, 48]. To tackle this problem, the solution proposed by Yun (2004) is followed. This is simple but robust: a linearisation around $E\left(X\right)\beta$ using a set of weights from a first-order Taylor linearisation around Eq. (6) [47]. This allows the contribution of the covariates to ${\Delta }_{X}^{\mu }$ and ${\Delta }_{\beta }^{\mu }$ to be seen as relative contributions fixed at the level of the linear predictor [40]. For this, let $\widehat{E}(X|G=g={\overline{X} }^{g})$ and $\widehat{E}(F(X\beta )|G=g={\stackrel{-}{F\left(X\beta \right)}}^{g}$. Thus, the aggregate decomposition can be expressed as:

7 $$\begin{array}{l}\widehat\Delta^\mu=\left\{\overline{F{(X\widehat\beta^0)}^0}-\overline{F{(X\widehat\beta^0)}^1}\right\}+\left\{\overline{F{(X\widehat\beta^0)}^1}-\overline{F{(X\widehat\beta^1)}^1}\right\}\\ \;\;\;\;\;\;=\widehat\Delta_X^\mu+\widehat\Delta_\beta^\mu\end{array}$$

The individual contribution of each covariate to the characteristics and coefficients effects can be estimated as [40]:

8 $${\widehat{\Delta }}_{X,{X}_{k}}^{\mu }=\frac{\left({\overline{X} }_{k}^{0}-{\overline{X} }_{k}^{1}\right){\widehat{\beta }}_{k}^{0}}{\left({\overline{X} }^{0}-{\overline{X} }^{1}\right){\widehat{\beta }}^{0}}{\widehat{\Delta }}_{X}^{\mu }$$

and

9 $${\widehat{\Delta }}_{\beta ,{\beta }_{k}}^{\mu }=\frac{{\overline{X} }_{k}^{1}({\widehat{\beta }}_{k}^{0}-{\widehat{\beta }}_{k}^{1})}{{\overline{X} }^{1}({\widehat{\beta }}^{0}-{\widehat{\beta }}^{1})}{\widehat{\Delta }}_{\beta }^{\mu }$$

such that ${\sum }_{i=1}^{K}{\widehat{\Delta }}_{X,{X}_{k}}^{\mu }={\widehat{\Delta }}_{X}^{\mu }$ and ${\sum }_{i=1}^{K}{\widehat{\Delta }}_{\beta ,{X}_{k}}^{\mu }={\widehat{\Delta }}_{\beta }^{\mu }$. Thus, Yun (2004) proposes to approximate ${\widehat{\Delta }}^{\mu }$ by first evaluating the function $F(.)$ at the means of the covariates [47],

10 $${\widehat{\Delta }}^{\mu }\approx \left[F\left({\overline{X} }^{0}{\widehat{\beta }}^{0}\right)-F\left({\overline{X} }^{1}{\widehat{\beta }}^{0}\right)\right]+ \left[F\left({\overline{X} }^{1}{\widehat{\beta }}^{0}\right)-F\left({\overline{X} }^{1}{\widehat{\beta }}^{1}\right)\right]$$

and then linearising the differences around ${\overline{X} }^{0}{\widehat{\beta }}^{0}$ and ${\overline{X} }^{1}{\widehat{\beta }}^{1}$ using a first order Taylor expansion.

The linearisation and first order Taylor expansion is as follows:

11 $$\begin{array}{l}{\widehat{\Delta }}^{\mu }\approx \left[F\left({\overline{X} }^{0}{\widehat{\beta }}^{0}\right)-F\left({\overline{X} }^{1}{\widehat{\beta }}^{0}\right)\right]+ \left[F\left({\overline{X} }^{1}{\widehat{\beta }}^{0}\right)-F\left({\overline{X} }^{1}{\widehat{\beta }}^{1}\right)\right]+{R}_{M}\\ \;\;\;\;\;\;\approx \left[\left({\overline{X} }^{0}-{\overline{X} }^{1}\right){\widehat{\beta }}^{0}\right]{d}^{0}+ \left[{\overline{X} }^{1}\left({\widehat{\beta }}^{0}-{\widehat{\beta }}^{1}\right)\right]{d}^{1}+{R}_{M}+{R}_{T}\end{array}$$

where

12 $$\begin{array}{c}R_M=\left[\overline{F{(X\widehat\beta^0)}^0}-\overline{F{(X\widehat\beta^0)}^1}\right]+\left[\overline{F{(X\widehat\beta^0)}^1}-\overline{F{(X\widehat\beta^1)}^1}\right]-\\ \;\;\;\;\;\;\;\;\;-\left[F\left(\overline X^0\widehat\beta^0\right)-F\left(\overline X^1\widehat\beta^0\right)\right]-\left[F\left(\overline X^1\widehat\beta^0\right)-F\left(\overline X^1\widehat\beta^1\right)\right]\end{array}$$

and

13 $$\begin{array}{lc}{R}_{T}=\left[F\left({\overline{X} }^{0}{\widehat{\beta }}^{0}\right)-F\left({\overline{X} }^{1}{\widehat{\beta }}^{0}\right)\right]+ \left[F\left({\overline{X} }^{1}{\widehat{\beta }}^{0}\right)-F\left({\overline{X} }^{1}{\widehat{\beta }}^{1}\right)\right]-\\ \;\;\;\;\;\; -\left[{(\overline{X} }^{0}-{\overline{X} }^{1}){\beta }^{0}\cdot {d}^{0} \right]-\left[{\overline{X} }^{1}({\beta }^{0}-{\beta }^{1})\cdot {d}^{1}\right]\end{array}$$

where ${d}^{g}$ represents the first derivative of $F\left({\overline{X} }^{g}{\widehat{\beta }}^{g}\right)=\frac{\partial F({\overline{X} }^{g}{\widehat{\beta }}^{g})}{\partial ({\overline{X} }^{g}{\widehat{\beta }}^{g})}$. Yun (2004) also mentions that ${R}_{M}$ and ${R}_{T}$ are approximation residuals from the evaluation of the function $F(.)$ at the means values and the linearisation [47]. After this, the set of weights for the explained part can be calculated as:

14 $${W}_{{\Delta }_{Xk}}=\frac{\left({(\overline{X} }_{k}^{0}-{\overline{X} }_{k}^{1}){\widehat{\beta }}_{k}^{0}){d}^{0}\right)}{\left({(\overline{X} }^{0}-{\overline{X} }^{1}){\widehat{\beta }}^{0}){d}^{0}\right)}=\frac{{(\overline{X} }_{k}^{0}-{\overline{X} }_{k}^{1}){\widehat{\beta }}_{k}^{0}}{{(\overline{X} }^{0}-{\overline{X} }^{1}){\widehat{\beta }}^{0}}$$

and for the unexplained part as:

15 $${W}_{{\Delta }_{\beta k}}=\frac{\left({(\widehat{\beta }}_{k}^{0}-{\widehat{\beta }}_{k}^{1}){\overline{X} }_{k}^{1}){d}^{1}\right)}{\left({(\widehat{\beta }}^{0}-{\widehat{\beta }}^{1}){\overline{X} }^{1}){d}^{1}\right)}=\frac{{(\widehat{\beta }}_{k}^{0}-{\widehat{\beta }}_{k}^{1}){\overline{X} }_{k}^{1}}{{(\widehat{\beta }}^{0}-{\widehat{\beta }}^{1}){\overline{X} }^{1}}$$

such that,

16 $${W}_{{\Delta }_{Xk}}={W}_{{\Delta }_{\beta k}}=1$$

The weights, ${W}_{{\Delta }_{XK}},$ show the contribution of the *k*^*th*^ variable to the linearisation of the explained part according to the magnitude of the mean group difference and accounting for the reference group's effect [48]. Thus, this detailed decomposition using weights is path invariant. The decomposition can be expressed in terms of the overall components as a sum of weighted sums of the unique contributions, as:

17 $${\overline Y}_A-{\overline Y}_B=E+U \;\;\;\;\;\;\;\;\;\;\;\;\;\;\;=\sum\nolimits_{k=1}^KW_{\Delta_{Xk}}E+\sum\nolimits_{k=1}^KW_{\Delta_{\beta k}}U=\sum\nolimits_{k=1}^KE_k+\sum\nolimits_{k=1}^KU_k$$

Jann (2018) warns that if the volume of data is in highly nonlinear regions of $F\left(.\right)$, or differences in coefficients or means are large, the approximation could be poor [40].

#### Additional Analysis

To provide additional policy-related evidence about ethnic disparities in the COVID-19 outcomes, the OB decomposition is carried out by stratifying the sample according to the type of public health hospital that provided care. For this, the public institutions that compose the Mexican health system are grouped in two categories: those that belong to the Ministry of Health (Federal and State hospitals) and those that work under a social security scheme (IMSS, ISSSTE, PEMEX, SEDENA and SEMAR). By doing this, it can be further tested whether receiving healthcare in a public hospital managed by the Ministry of Health or social security institutions matters more for ethnic differences. For all the OB decomposition estimations, levels of uncertainty are reported using bootstrapped standard errors based on 1**,**000 replications with replacement.

## Results

### Descriptive Analysis

Table 1 shows differences across both groups in individual characteristics and contextual circumstances along with the differences in mean values and the *p*-value associated with the test of mean differences. There are statistically significant differences across non-indigenous and indigenous people for all the covariates. On average, the Indigenous group had a higher age and proportion of women. The proportion of people with underlying health conditions is higher for non-Indigenous people. Concerning medical care, Table 1 shows that, non-indigenous people waited slightly less time than indigenous people to be tested for COVID-19. The proportion of Indigenous people that received care in hospitals managed by the health secretariat (SSA) was higher (0.77) than the proportion in the non-Indigenous population (0.65). In terms of the contextual circumstances, Table 1 shows that Indigenous people faced worse conditions in terms of medical infrastructure, higher levels of marginalisation and population dispersion. For example, by January 2020, the average density of medical offices and hospital beds in municipalities where non-Indigenous people lived was higher than where Indigenous people lived. Marginalisation and population dispersion were also lower in non-Indigenous municipalities.

**Table 1:** Ethnic differences in individual characteristics and contextual circumstances

	N all sample	Mean all sample	N Non-Indigenous	Mean Non-Indigenous	N Indigenous	Mean Indigenous	Diff	*p*-val
Individual-level characteristics								
* Demographics*								
Age (years)	4,827,684	41.7	4,796,427	41.82	31,257	45.07	-3.25	0.00
Women	4,829,071	0.48	4,797,799	0.48	31,272	0.51	-0.02	0.00
* Comorbidities*								
COPD	4,820,124	0.98	4,788,933	0.99	31,191	0.97	0.02	0.00
Asthma	4,820,318	0.97	4,789,118	0.97	31,200	0.97	0	0.02
Immunosuppression	4,819,928	0.99	4,788,734	0.99	31,194	0.99	0	0.00
Renal disease	4,820,287	0.98	4,789,092	0.99	31,195	0.98	0.01	0.00
Pneumonia	4,822,830	0.91	4,791,666	0.91	31,164	0.82	0.09	0.00
Other Comorbidities	4,813,370	0.98	4,782,224	0.98	31,146	0.98	0	0.01
* Non-Communicable Diseases*								
Diabetes	4,818,975	0.88	4,787,783	0.89	31,192	0.84	0.05	0.00
Hypertension	4,819,960	0.84	4,788,761	0.85	31,199	0.83	0.02	0.00
Cardiovascular disease	4,820,170	0.98	4,788,970	0.98	31,200	0.98	0	0.00
* Risky Behaviours*								
Smoking	4,819,646	0.91	4,788,446	0.91	31,200	0.94	-0.03	0.00
Obesity	4,820,588	0.87	4,789,380	0.87	31,208	0.86	0.01	0.00
* Medical Attention*								
Wait- time test (days)	4,829,071	3.38	4,797,799	3.44	31,272	3.51	-0.06	0.00
Health secretariat	4,718,808	0.32	4,687,863	0.65	30,945	0.77	-0.12	0.00
Social security	4,718,808	0.67	4,687,863	0.35	30,945	0.23	0.12	0.00
Contextual circumstances								
* Medical Infrastructure*								
Medical offices den	4,828,549	5.34	4,797,294	5.31	31,255	3.45	1.85	0.00
Hospital beds den	4,828,549	15.56	4,797,294	15.52	31,255	9.34	6.18	0.00
* Municipal Marginalisation*								
MM index	4,829,023	0.92	4,797,751	0.93	31,272	0.86	0.07	0.00
* Population dispersion*								
Percentage of urban localities in a municipality	4,829,023	28.35	4,797,751	28.11	31,272	15.15	12.95	0.00

Analysis period Jan 2020–March 2021. *N* sample size. *Diff* raw difference. Two-sided *p*-value of the test for differences in means. *Den.* density. *MM* municipal marginalisation, higher values indicate lower marginalisation

Table 2 shows the raw proportion of people hospitalised and dead, including those that died prematurely (early mortality), due to COVID-19 before the national vaccination campaign. The table shows the total number of non-Indigenous and Indigenous people and the proportion of individuals, of each group, hospitalised and dead as well as the difference in these proportions. For example, -0.120 (sixth column, first row) is the result of the difference between the proportion of non-Indigenous people and the proportion of Indigenous people hospitalised (0.129–0.249). This difference indicates that the proportion of hospitalisations was 0.12 greater among Indigenous than non-Indigenous people. The negative sign shows that Indigenous people were more affected than non-Indigenous by COVID-19. The p-value associated with a test of differences in proportions is displayed in the last column and indicates that differences are statistically significant. This table validates the application of the decomposition method since an ethnic gap exists.

**Table 2:** Ethnic differences in proportions of people hospitalised, and dead due to Covid-19 in Mexico

Outcomes	All sample	Prop	N Non-Indigenous	Prop. Non-Indigenous	N Indigenous	Prop Indigenous	Diff	*p*-val
People hospitalised	4,829,071	0.13	4,797,799	0.129	31,272	0.249	-0.120	0.000
People dead within 5 days	4,712,088	0.02	4,682,129	0.023	29,959	0.050	-0.027	0.000
People dead	4,829,071	0.05	4,797,799	0.051	31,272	0.098	-0.048	0.000

Analysis period Jan 2020–March 2021. *N* sample size. *Prop.* proportion. *Diff* raw difference. A two-sided *p*-value of the test for differences in proportions is displayed in the last column

Results from the logit regression models are found in Table A2. Overall, these models show the expected direction in the coefficients, e.g., a positive relationship between having underlying health conditions and being hospitalised or dying due to COVID-19 or an inverse relationship between medical office density and population dispersion and a lower likelihood of a negative health outcome.

### Decomposition Results

#### Aggregate Decomposition

Both tables, Tables 2 and 3, show the same information regarding the average health outcome for each group, ${\overline{Y} }^{g}$, *g* = 0,1. The outcomes take binary values and thus, both tables show the proportion of Indigenous and non-Indigenous people hospitalised and dead. However, Table 3 also shows the results of the average gap decomposition.

**Table 3:** Aggregate Oaxaca decomposition

	Hosp.	%	E. Deaths	%	Deaths	%
Non-Indigenous	0.126***		0.023***		0.050***	
	(0.00)		(0.00)		(0.00)	
Indigenous	0.245***		0.049***		0.097***	
	(0.00)		(0.00)		(0.00)	
Mean Difference	-0.119***		-0.027***		-0.047***	
	(0.00)		(0.00)		(0.00)	
Explained	-0.092***	77.2***	-0.022***	82.5***	-0.039***	83.6***
	(0.00)	(1.92)	(0.00)	(2.90)	(0.00)	(2.43)
Unexplained	-0.027***	22.8***	-0.005***	17.5***	-0.008***	16.4***
	(0.00)	(1.92)	(0.00)	(2.90)	(0.00)	(2.43)
N	4,688,278	4,688,278	4,575,481	4,575,481	4,688,278	4,688,278

*Hosp. *Hospitalisations. *E. *Early. Bootstrapped standard errors in parenthesis (1,000 replications). Models fitted using an ANOVA-type normalisation and weights from a first-order Taylor linearisation. % Share of each component to the overall gap. + *p* < 0.1, * *p* < 0.05, ** *p* < 0.01, *** *p* < 0.001

While the explained component, which depicts the extent of legitimate inequalities due to differences in observable characteristics, accounts for most of the average difference, 77.2% for hospitalisations, 82.5% for COVID-related early deaths and 83.6% for overall deaths. The unexplained component, which proxies illegitimate disparities, accounts for approximately 22.8%, 17.5% and 16.4% of the ethnic gap, respectively.

#### Detailed Decomposition

Table 4 shows the absolute and relative contributions of each sub-set of variables to the explained and unexplained components, respectively. Relative contributions are shown as a percentage of the overall difference. Positive contributions indicate that if the distribution of a characteristic is swapped between Indigenous and non-Indigenous people, a reduction in the ethnic gap would be expected. Likewise, a negative contribution indicates that if the counterfactual is observed, the ethnic gap is expected to increase.

**Table 4:** Detailed Oaxaca decomposition

	Hosp.	SE	E. Deaths	SE	Deaths	SE
*Explained component*						
Demographics	-0.006***	(0.00)	-0.007***	(0.00)	-0.016***	(0.00)
%	5.383***	(0.42)	25.733***	(1.98)	33.303***	(1.50)
Comorbidities	-0.049***	(0.00)	-0.011***	(0.00)	-0.023***	(0.00)
%	40.678***	(0.86)	42.015***	(2.44)	48.462***	(2.16)
NCDs	-0.004***	(0.00)	-0.001***	(0.00)	-0.001**	(0.00)
%	3.738***	(0.44)	2.931***	(0.52)	2.727***	(0.80)
Risky Behaviours	-0.001**	(0.00)	-0.001*	(0.00)	-0.001**	(0.00)
%	1.141**	(0.43)	2.230*	(1.10)	2.147***	(0.64)
Med. Attention	0.018***	(0.00)	0.002***	(0.00)	0.006***	(0.00)
%	-15.432***	(0.54)	-8.805***	(2.12)	-12.807***	(1.64)
Health Infrastructure	0.001	(0.00)	-0.004***	(0.00)	-0.004*	(0.00)
%	-0.527	(2.64)	13.400***	(3.13)	7.424*	(2.94)
Marginalisation	-0.036***	(0.00)	0.002	(0.00)	0.005**	(0.00)
%	30.024***	(1.99)	-6.905	(6.35)	-11.423**	(3.60)
Pop. Disp.	-0.008**	(0.00)	-0.002 +	(0.00)	-0.003 +	(0.00)
%	6.431**	(2.04)	7.588 +	(4.56)	5.850 +	(3.36)
Temporality	-0.007***	(0.00)	-0.001**	(0.00)	-0.004***	(0.00)
%	5.729***	(0.80)	4.310**	(1.39)	7.975***	(2.15)
*Unexplained component*						
Demographics	0.031***	(0.00)	0.004***	(0.00)	0.006***	(0.00)
%	-25.661***	(1.70)	-13.685***	(3.08)	-13.241***	(2.10)
Comorbidities	-0.013*	(0.01)	-0.000	(0.00)	-0.002	(0.00)
%	11.223*	(4.66)	1.649	(5.07)	4.952	(5.57)
NCDs	0.000	(0.00)	-0.000	(0.00)	-0.001	(0.00)
%	-0.410	(2.56)	0.431	(2.52)	1.241	(1.51)
Risky Behaviours	-0.002	(0.00)	0.000	(0.00)	0.000	(0.00)
%	2.038	(1.30)	-0.148	(2.02)	-0.494	(1.29)
Med. Attention	-0.002***	(0.00)	-0.001	(0.00)	-0.001 +	(0.00)
%	1.615***	(0.33)	2.226	(1.43)	1.705 +	(0.90)
Health Infrastructure	-0.004 +	(0.00)	0.001 +	(0.00)	0.000	(0.00)
%	3.622 +	(2.11)	-2.329 +	(1.29)	-0.434	(0.84)
Marginalisation	0.045***	(0.01)	-0.011**	(0.00)	-0.009**	(0.00)
%	-37.321***	(7.15)	41.226**	(13.35)	18.392**	(6.90)
Pop. Disp.	0.002	(0.00)	0.000	(0.00)	-0.000	(0.00)
%	-1.262	(1.48)	-0.348	(1.42)	0.224	(1.00)
Temporality	0.154***	(0.04)	0.025	(0.43)	0.048	(0.62)
%	-129.456***	(36.66)	-91.965	(1616.82)	-102.582	(1315.22)
Intercept	-0.237***	(0.05)	-0.021	(0.43)	-0.050	(0.62)
%	198.449***	(38.21)	80.445	(1609.48)	106.579	(1319.47)
N	4,688,278		4,575,481		4,688,278	

*Hosp. *Hospitalisations. *E*. Early. *SE *Standard error. Bootstrapped standard errors in parenthesis (1,000 replications). Models fitted using an ANOVA-type normalisation and weights from a first-order Taylor linearisation. % Share of each component to the overall gap. + p < 0.1, * p < 0.05, ** p < 0.01, *** p < 0.001

Demographics and comorbidities positively contribute to closing the explained ethnic gap in all outcomes, while medical attention contributes negatively to increasing disparities. The presence of comorbidities is the main driver of the explained ethnic differences. This means that if Indigenous were equal to non-Indigenous in the distribution of their comorbidities, the ethnic gap in hospitalisations, early deaths and deaths would be expected to be reduced by 40.6%, 42%, and 48.4%., respectively. The type of health institution where individuals received medical attention is a factor that increases the indigenous/non-Indigenous health gap. If Indigenous people were treated in the same health institutions where non-Indigenous people received care, the ethnic difference in the COVID-19 outcomes would have increased by 15.4%, 8.8%, and 12.8%.

Differences in the intercepts (baseline logs) are the main driver of discrimination, although these estimations are statistically significant for hospitalisations only. Municipal marginalisation is the second driver of the unexplained part of the gap. It contributes negatively to the gap in hospitalisations and positively to ethnic differences in deaths. This indicates that while it represents a key factor for illegitimate disparities against Indigenous people in hospitalisations, differences in the effect of coefficients between groups contribute to reducing disparities in COVID-related deaths.

### Decomposition Results by Type of Health Provider

Table A3 depicts the results from the aggregate decomposition stratified by the Ministry of Health and Social Security health institutions. As in the general decomposition model, most of the health gap is due to observable characteristics (79–86%) and there is a remaining unexplained part of around 21–14%. Overall, decomposing the mortality gap, both early and general, by type of health provider shows similar results.

With respect to the detailed decomposition, Table A4 shows that individual-level characteristics such as demographics and comorbidities are the main drivers of the explained components. Notwithstanding, contextual circumstances such as population dispersion and socioeconomic municipal marginalisation were particularly relevant for those individuals treated in hospitals managed by the Ministry of Health. The detailed decomposition of the unexplained component among those that received care in Social Security institutions show a lot of uncertainty as the standard errors are very large, which hinders a robust interpretation of the estimations.

## Discussion

Using administrative and census data on COVID-19, this study examined discrimination in hospitalisations, early deaths, and deaths due to COVID-19 between Indigenous and non-Indigenous people in Mexico. This paper contributes to the ongoing literature about ethnic health disparities by analysing the extent of their illegitimacy and the contribution of the relevant factors to their amplification or reduction. This study offers novel evidence about the presence of ethnic discrimination, understood as the exercise of formal or informal practices that systematically produce unjustified unequal treatment between Indigenous and non-Indigenous people.

Results indicate that despite most part of the gap in COVID-19 health outcomes being explained by differences in observable characteristics, the remaining unexplained part could be attributable to discrimination against indigenous people. Differences in age, sex, underlying health conditions and exposure to socioeconomic marginalisation are key factors that *potentially* justify the outsized effect of the COVID-19 pandemic on Indigenous individuals. Notwithstanding, this is debatable. Particularly those concerning to different levels of marginalisation across municipalities. In principle, high and heterogenous levels of marginalisation across areas are negative to social welfare. Thus, to observe that differences in marginalisation levels unfavourably impact indigenous communities is illegitimate and implies acknowledging two situations, a) the evidence about the unequal treatment in the allocation of public funds to States and Municipalities where Indigenous populations live and, b) the limitations of the federal political system that prevails in Mexico. A pioneering study about public spending in Mexico found that unequal allocation of resources is one hidden form of indirect or structural discrimination since public spending does not consider any kind of compensation or prioritising criteria in the formulae used to assign public resources [27]. Interconnected to this is the fact that the political federal arrangement has led to different levels of efficiency, efficacy, and quality in the provision of health services across the Mexican States. Mexican federalism and some asymmetrical traits have deepened social inequalities [49]. Historically, States with a higher presence of Indigenous settlements have shown relatively fewer health facilities along with low levels of quality of healthcare services [13–15, 50]. This highlights the need to consolidate a coordinating and responsive federal system that can guarantee universal health insurance coverage, access to basic medical care and public basic services for all citizens, regardless of their ethnicity or postcode.

On the question of whether there is structural discrimination in health outcomes against Indigenous people in Mexico, this study found evidence to support this claim. By disentangling the ethnic health gap, around 22–16% of the differences were found to be unjustifiable via individual, contextual circumstances, and temporal effects. Previous research evidence has corroborated that indigenous populations in Mexico experience systematic discrimination in health. For example, a comprehensive analysis of public expenditure in Mexico revealed that Indigenous populations showed an average level of exclusion in public spending of around 85% in 2010/11, 2011/12, and 2012/13 fiscal years [27]. Respecting health, there is evidence about Indigenous people not utilising primary health care services due to the lack of confidence, mistreatment, unavailability, and facility’s remoteness [13]. A similar study to this analysis found that disparities in effective coverage of maternal healthcare between Indigenous and non-Indigenous women were explained in 33.29% by structural discrimination. This evidence is concerning considering the high-level agreements that the Mexican government has signed to protect the aspirations of Indigenous people to develop and maintain their identities, languages and religions while exercising their fundamental human rights to the same extent as the rest of the population. With regards to health, these agreements declare that social security and health services should be extended progressively to reach full coverage, that the delivery of health services should be community-based, and that the health system should prioritise the delivery of primary health care services [51].

Moreover, the current study also found that differences in the quality of care within the public sphere of the health system exist and affects to a higher extent Indigenous people. Higher levels of discrimination were observed in Social Security institutions compared to hospitals managed by the Ministry of Health. The main contributor to this discrimination was the levels of municipal marginalisation. If the levels of marginalisation across municipalities were equalised, the ethnic gap would decrease by 58% in hospitalisations, 36% in early deaths and 31% in deaths (general). These results are consistent with a prior study about the higher probability to die due to COVID-19 among people treated in the Mexican Social Security Institute [37] compared to other health provider institutions. Despite this latter study showing unconditional probabilities, it offers insights into the existence of structural differences in the hospital infrastructure, equipment availability and training of the staff as well as the use of care protocols and that the pandemic only exhibited these deep-rooted inequalities [36].

This analysis is not without limitations. One of these concerns the administrative dataset used. Since barriers to accessing the health system exist, many people died in their homes and, therefore, were not registered in the SINAVE. This has led to at least two main issues not being tackled in this study. On the one side, there is selection bias. As COVID-19 data do not come from a random selection of individuals, there might be a sub-representation of the groups. This is particularly concerning for Indigenous people, who tend to face higher limitations in accessing the health system in Mexico. On the other, all COVID-related deaths that occurred outside of the health system are not included in this analysis. Again, there is evidence that this phenomenon affected particularly more Indigenous people [52]. An implication of these issues is that the decomposition estimations are downward biased due to the partial observability of COVID-19 cases among Indigenous people. Hence, the decomposition results can be interpreted as lower-bound estimates of the true levels of illegitimate ethnic inequity. Another caution when interpreting these results is that, although the model included a comprehensive number of variables at the individual and contextual levels, there remains the potential omission of relevant variables that could be related to the outcomes and that are currently not included in the estimations. This latter precludes a causal interpretation of the results.

One final comment is that due to restricted data availability, this paper used a language-speaking-based ethnicity identification. This is a point worth discussing. In Mexico, there are several institutional definitions to identify indigenous groups. On the one side, the National Commission for the Development of the Indigenous Peoples of Mexico (CDI) defines indigenous people as those individuals who are part of a household where the head, spouse or one of the ascendants declares to be a speaker of an indigenous language. On the other, INEGI defines indigenous people as those individuals above three years old that speak an indigenous language. The use of different concepts can potentially lead to deliberatively excluding indigenous people from different spheres, ranging from population estimations to policy-making decisions [14, 53–55]. Thus, this is another source of underestimation of sample size for indigenous people since there might have been individuals that, although ascribed to an indigenous household and self-identified as indigenous, were not registered as such in the dataset. It might be relevant in future works to use a different dataset—for example, the excess mortality dataset, which includes all deaths in the country and identifies indigenous people according to their membership to an indigenous household- to re-estimating the magnitude of the gap. Despite these limitations, this work has thrown up many research questions in need of further investigation as it would be determining the dynamics of ethnic discrimination in COVID-19 by extending the cross-sectional decomposition analysis to mean group differences over different points in time or by extending the period of analysis to the vaccination period. This latter would provide the possibility to test the hypothesis of structural discrimination against Indigenous people embedded in decisions about priority groups.

In sum, these findings reveal the existence of public and governmental mechanisms that generate and reproduce ethnic health inequities. This study contributes to the wider discussion about health-related discriminatory practices against indigenous people which jeopardise the capacity of multi-ethnic countries to achieve social justice in health. The challenge for Mexico, and similar countries, relies in great part on overcoming the barriers to health access and heterogenous quality of care that a fragmented health system entails. In this context, this work also calls for re-considering governmental commitments to focalise and prioritise the welfare of Indigenous people in their decision-making processes.

## Conclusion

All in all, this analysis has identified that Indigenous people in Mexico faced worse COVID-19 outcomes than the general population and unveiled the existence of systematic barriers that affect Indigenous groups in a distinct and exclusionary manner. Indigenous populations in Mexico observe higher levels of socioeconomic deprivation and limited healthcare access which contribute to inadequate healthcare utilisation and, therefore, increase long-term illnesses. Relentless ethnic health disparities have been identified and indeed acknowledged; however, they remained systematically ignored in policy-making decisions. Since COVID-19 is exacerbating the pre-existing, deep-rooted and longstanding health inequalities between Indigenous and non-Indigenous people, it is imperative to design programmes that prioritise and target Indigenous people and to enhance the current social and health policies if the disproportionate impact of this and future epidemics is aimed to be mitigated.

## Supplementary Information

Below is the link to the electronic supplementary material.

Supplementary file1 (PDF 111 KB)

Supplementary file2 (DOCX 21 KB)

Supplementary file3 (DOCX 28 KB)

Supplementary file4 (DOCX 17 KB)

Supplementary file5 (DOCX 28 KB)

Supplementary file6 (PNG 247 KB)

Supplementary file7 (PNG 610 KB)

## Data Availability

The data that support the findings of this study are available in the public domain.

## Footnotes

1 See Fig. A1.

2 A similar approach was followed by Mancilla et al. (2020) [30] in their modelling about COVID-19 deaths in Mexico.

3 The Mexican health system is primarily divided into public/private spheres, but within the public system, six institutions provide health and social care. These institutions are the Mexican Social Security Institute (IMSS); the Civil Service Social Security and Services Institute (ISSSTE); Health Ministry programmes, such as Seguro Popular or INSABI (the Seguro Popular programme was targeted for people with no health insurance and started in 2003. A reform took place in 2019 and the Seguro Popular programme disappeared and the Institute of Health for Welfare (INS- ABI) was created to substitute Seguro Popular); the state-owned petroleum company: Mexican Petroleum (PEMEX); the Secretariat of National Defence (SEDENA) and the Secretariat of Navy (SEMAR). Membership in these institutions depends on people’s jobs. Examples of affiliations include people working in the informal sector enrolled in the Seguro Popular programme; private company workers affiliated to the IMSS; secondary-level teachers working in a public school are insured by the ISSSTE. Workers of PEMEX, SEDENA or SEMAR receive health and social care in their institutions. Senior public servants tend to have major private medical insurance [34, 35].

4 This description follows the notation of Jann (2008) [40]

5 There is also the useful property of the logit regression in that by including a constant term, the average of the predicted probabilities must equal the proportion of the sample [42].

6 An additional and relevant point regarding the interpretation of this component, although beyond the scope of this work, is that of Fortin et al. (2011) [43] who state and explain the link between this decomposition method with the impact evaluation literature, which is that the unexplained component can be interpreted as the population treatment effect on the treated (PATT) if selection on observables is assumed and holds for the identification of treatment effects. This vision has also been shared by Sloczyński (2015, 2020) [44, 45]. Jann (2008) [46] also mentions that the unexplained part captures all the potential effects of differences in unobserved variables.
