# Supplementary material for: Socioeconomic Inequalities and Ethnic Discrimination in COVID-19 Outcomes: the Case of Mexico
Source: J Racial Ethn Health Disparities. 2023 Apr 11;11(2):900–12. doi: 10.1007/s40615-023-01571-z (PMC10089566; doi:10.1007/s40615-023-01571-z)
Supplement: Supplementary file 1 — Supplementary file1 (PDF 111 KB) [file 40615_2023_1571_MOESM1_ESM.pdf]

Socioeconomic inequalities and ethnic discrimination in COVID-19 outcomes. The case of Mexico

Supplementary Material A

COVID-19 procedures and data collection

The testing procedure was as follows: people who have symptoms and sought out a test arrive at the health unit (this assumes that people are physically capable of going to their regular health unit). Once in the health facility, the general practitioner (GP) screens the patient and decides if the patient meets the inclusion criteria to be tested for COVID-19. If patients are tested, GPs capture information about their medical history, the current date, and thedate when the patient first showed a symptom. This information is recordedin an online platform called SINAVE (National Epidemiological Surveillance System). Cases in the dataset represent both ambulatory (outpatient) andhospitalised (inpatient) individuals. Swabs are obtained from outpatients and samples are sent to the nearest Laboratory of Respiratory Virus (InDRE, in Spanish). This process could take up to four days. If the case is positive, there are two potential paths to follow which depends on the health status of the patient.If the person is clinically assessed and diagnosed as with a mild to moderate infection, the person can remain at home and be remotely monitored. Follow- up of all suspected COVID-19 cases and ambulatory patients is done by the responsible healthcare professional of every Local Health Jurisdiction. Thisperson is also in charge of uploading the data into SINAVE. Due to collection procedures, a patient who is tested more than once in different jurisdictions and at different points in time may lead to duplicate records as there is no unique identification variable available to identify individual patients.

A patient clinically diagnosed with a complicated to severe infection (when the patient has difficulties with breathing or hypoxemia) is admitted to a specialised COVID-19 hospital. In these hospitals, patients immediately receive drug and oxygen treatment. If patients do not respond favourably to the treatment, they can be admitted to the intensive care unit (ICU). It is also possible that patients who never asked for a test when they first felt symptoms could arrive at a hospital seeking medical attention, without any previous test

or clinical record. In this scenario, patients are rapidly screened and if the test is positive the patient is admitted to a Covid-hospital; if not, the patient is referred to another hospital to receive care. In the case of patients that for somereason are already intubated, bronchoalveolar lavage sample is obtained and tested for COVID-19. If an inpatient died due to suspected COVID-19, lung biopsies are obtained from an autopsy. Reporting of deaths is obligatory and must be done in less than 48 hours after occurrence. If patients are not ableto give details about their medical history, this is retrieved from records. All these data are undertaken by accredited hospital epidemiologists and uploadedin the SINAVE.
