# Supplementary material for: Socioeconomic Inequalities and Ethnic Discrimination in COVID-19 Outcomes: the Case of Mexico
Source: J Racial Ethn Health Disparities. 2023 Apr 11;11(2):900–12. doi: 10.1007/s40615-023-01571-z (PMC10089566; doi:10.1007/s40615-023-01571-z)
Supplement: Supplementary file 2 — Supplementary file2 (DOCX 21 KB) [file 40615_2023_1571_MOESM2_ESM.docx]

**Table A1** Variables definition and sources

| **Group** | **Sub-group** | **Definition** | **Type of variable** | **Source** |
| --- | --- | --- | --- | --- |
| **Individual-level characteristics** | | | | |
| **Demographics** | Sex | Sex of the individual. 1 if female, 0 male | Binary | SINAVE |
|  | Age | Age Individual years of age | Continuous |  |
| **Underlying Health Conditions** | Pneumonia* | 1 if the patient has a diagnosis of pneumonia, 0 otherwise | Binary |  |
|  | Hypertension* | 1 if the patient has a diagnosis of hypertension, 0 otherwise |  |  |
|  | Diabetes* | 1 if the patient has a diagnosis of diabetes, 0 otherwise |  |  |
|  | COPD* (Chronic obstructive pulmonary disease) | 1 if the person has a diagnosis of COPD, 0 otherwise |  |  |
|  | Asthma* | 1 if the patient has a diagnosis of asthma, 0 otherwise |  |  |
|  | Immunosuppression* | 1 if the patient has immunosuppression, 0 otherwise |  |  |
|  | Renal disease* | 1 if the patient has a diagnosis of renal disease, 0 otherwise |  |  |
|  | Cardiovascular disease* | 1 if the patient has a diagnosis of cardiovascular disease, 0 otherwise |  |  |
|  | Other underlying health condition + | Other comorbidities |  |  |
| **Risky health**  **behaviours** | Obesity* | To be obese. 1 if the patient has obesity, 0 otherwise. |  |  |
|  | Smoking | To smoke. 1 if the patient smokes regularly, 0 otherwise |  |  |
| **Medical attention** | Testing waiting time | Number of days the person waited to get tested since the first symptom | Continuous |  |
|  | Type of health provider | 1 if the patient received health attention in social security institutions: Mexican Social Security Institute (IMSS, in Spanish); Civil Service Social Security and Services Institute (ISSSTE, in Spanish); Hospitals owned and managed by the state-owned petroleum company “Mexican Petroleum” (PEMEX, in Spanish); Hospitals owned and managed by the Secretariat of National Defence (SEDENA, in Spanish) or Hospitals owned and managed by the Secretariat of the Navy (SEMAR, in Spanish).  0 otherwise. | Binary |  |
|  |  | 1 if the patient received health attention in health secretariat institutions: Federal and State Ministry of Health-owned hospitals (these hospitals provide health services to people enrolled in the INSABI programme, formerly known as “Seguro Popular”).  0 otherwise. | Binary |  |
| **Contextual circumstances** | | | | |
| **Health infrastructure** | Medical offices density | Number of medical offices per 10,000 inhabitants by January 2020 | Continuous | DGIS |
|  | Hospital beds density | Number of hospital beds per 10,000 inhabitants by January 2020 | Continuous |  |
| **Socioeconomic marginalisation** | Municipal marginalisation index | Summary measure that indicates the level of socioeconomic deficiencies in a municipality in terms of education, housing, distribution of the population and monetary income such as the percentage of the population above 15 years of age that is illiterate, percentage of the population above 15 years of age without schooling, percentage of households without a toilet or drainage, percentage of households without electricity, percentage of households without piped water, percentage of households with dirt floors, percentage of households with overcrowding, percentage of the population that live in localities with less than 5,000 inhabitants and, percentage of the employed population earning up to two minimum salaries.  This is an index bounded between 0 and 1. Lower values depict higher levels of marginalisation. The 2020 marginalisation index was used. | Continuous | CONAPO-INEGI |
| **Population dispersion** | Urbanity | Percentage of urban localities in each municipality | Continuous | INEGI |
| **Temporality** | Date | Month and year | Binary | SINAVE |

## Notes: * There is no clinical definition available in the dataset for these variables. +No additional information was provided
