# Supplementary material for: Socioeconomic Inequalities and Ethnic Discrimination in COVID-19 Outcomes: the Case of Mexico
Source: J Racial Ethn Health Disparities. 2023 Apr 11;11(2):900–12. doi: 10.1007/s40615-023-01571-z (PMC10089566; doi:10.1007/s40615-023-01571-z)
Supplement: Supplementary file 3 — Supplementary file3 (DOCX 28 KB) [file 40615_2023_1571_MOESM3_ESM.docx]

**Table A2** Results from Logit regression models

|  | **Hosp.** |  | **Hosp.** |  | **E. Deaths** |  | **E. Deaths** |  | **Deaths** |  | **Deaths** |  |
| --- | --- | --- | --- | --- | --- | --- | --- | --- | --- | --- | --- | --- |
|  | *G_i_=0* |  | *G_i_=1* |  | *G_i_=0* |  | *G_i_=1* |  | *G_i_=0* |  | *G_i_=1* |  |
| *Demographics* |  |  |  |  |  |  |  |  |  |  |  |  |
| Age (years) | 0.03*** | (0.00) | 0.01*** | (0.00) | 0.05*** | (0.00) | 0.04*** | (0.00) | 0.06*** | (0.00) | 0.05*** | (0.00) |
| Women | 0.37*** | (0.00) | 0.13** | (0.04) | 0.44*** | (0.01) | 0.23*** | (0.07) | 0.52*** | (0.01) | 0.38*** | (0.05) |
| *Comorbidities* |  |  |  |  |  |  |  |  |  |  |  |  |
| COPD | -0.50*** | (0.01) | -0.28** | (0.11) | 0.00 | (0.02) | 0.19 | (0.12) | 0.03* | (0.01) | 0.29** | (0.10) |
| Asthma | 0.26*** | (0.01) | 0.12 | (0.12) | 0.26*** | (0.03) | 0.01 | (0.18) | 0.23*** | (0.02) | -0.07 | (0.14) |
| Immunosuppression | -1.00*** | (0.02) | -1.18*** | (0.15) | -0.37*** | (0.02) | -0.05 | (0.23) | -0.40*** | (0.02) | -0.14 | (0.18) |
| Renal disease | -1.22*** | (0.01) | -0.82*** | (0.12) | -0.72*** | (0.01) | -0.54*** | (0.14) | -0.68*** | (0.01) | -0.41*** | (0.12) |
| Pneumonia | -4.03*** | (0.01) | -3.82*** | (0.05) | -2.59*** | (0.01) | -2.69*** | (0.07) | -2.67*** | (0.01) | -2.69*** | (0.05) |
| Other Comorb.^a^ | -0.70*** | (0.01) | -0.52*** | (0.11) | -0.34*** | (0.02) | -0.57*** | (0.15) | -0.32*** | (0.01) | -0.41** | (0.13) |
| *Non-Communicable Diseases* | | | | | | | | | | | | |
| Diabetes | -0.63*** | (0.01) | -0.58*** | (0.05) | -0.43*** | (0.01) | -0.28*** | (0.07) | -0.38*** | (0.01) | -0.25*** | (0.06) |
| Hypertension | -0.29*** | (0.01) | -0.26*** | (0.05) | -0.17*** | (0.01) | -0.21** | (0.07) | -0.17*** | (0.01) | -0.16** | (0.06) |
| Cardio disease | -0.54*** | (0.01) | -0.61*** | (0.12) | 0.03+ | (0.02) | -0.04 | (0.15) | 0.11*** | (0.01) | 0.10 | (0.13) |
| *Risky Behaviours* |  |  |  |  |  |  |  |  |  |  |  |  |
| Smoking | 0.22*** | (0.01) | 0.30** | (0.09) | 0.17*** | (0.01) | 0.29* | (0.13) | 0.19*** | (0.01) | 0.25* | (0.10) |
| Obesity | -0.00 | (0.01) | 0.06 | (0.05) | -0.21*** | (0.01) | -0.36*** | (0.08) | -0.26*** | (0.01) | -0.38*** | (0.06) |
| *Medical Attention* |  |  |  |  |  |  |  |  |  |  |  |  |
| Wait time test (days) | 0.03*** | (0.00) | 0.03*** | (0.01) | 0.04*** | (0.00) | 0.03*** | (0.01) | 0.05*** | (0.00) | 0.04*** | (0.01) |
| Social security | 1.49*** | (0.00) | 1.17*** | (0.04) | 1.08*** | (0.01) | 0.38*** | (0.07) | 1.18*** | (0.01) | 0.56*** | (0.05) |
| *Medical Infrastructure* | | | | | | | | | | | | |
| MOD^b^ | -0.02*** | (0.00) | -0.01 | (0.01) | -0.02*** | (0.00) | -0.01 | (0.02) | -0.02*** | (0.00) | -0.02 | (0.01) |
| HBD^c^ | 0.00* | (0.00) | 0.01 | (0.00) | 0.00*** | (0.00) | -0.01 | (0.01) | 0.00*** | (0.00) | -0.00 | (0.00) |
| *Municipal Marginalisation* | | | | | | | | | | | | |
| MM^d^ index | -2.78*** | (0.07) | -3.91*** | (0.26) | -1.19*** | (0.13) | 0.51 | (0.44) | 0.06 | (0.10) | 0.84* | (0.35) |
| *Population dispersion* | | | | | | | | | | | | |
| Urban localities^e^ | -0.00*** | (0.00) | -0.00*** | (0.00) | -0.00*** | (0.00) | -0.00+ | (0.00) | -0.00*** | (0.00) | -0.00+ | (0.00) |
| *Temporality*^f^ |  |  |  |  |  |  |  |  |  |  |  |  |
| Feb 20 | 2.00*** | (0.06) | 2.33*** | (0.68) | 0.11 | (0.20) | 0.79 | (1.48) | 1.01*** | (0.14) | 1.89 | (1.23) |
| Mar. 20 | 0.73*** | (0.03) | 1.22*** | (0.34) | 0.99*** | (0.11) | 1.49 | (1.11) | 1.98*** | (0.09) | 2.15* | (1.09) |
| Apr. 20 | 0.38*** | (0.03) | 1.10*** | (0.28) | 2.15*** | (0.09) | 2.63* | (1.03) | 2.97*** | (0.08) | 3.57*** | (1.03) |
| May 20 | 0.20*** | (0.02) | 0.58* | (0.27) | 2.28*** | (0.09) | 2.73** | (1.03) | 3.04*** | (0.08) | 3.63*** | (1.03) |
| June 20 | -0.03 | (0.02) | 0.48+ | (0.27) | 2.11*** | (0.09) | 2.61* | (1.03) | 2.88*** | (0.08) | 3.48*** | (1.03) |
| July 20 | -0.19*** | (0.02) | 0.42 | (0.27) | 1.98*** | (0.09) | 2.68** | (1.03) | 2.73*** | (0.08) | 3.54*** | (1.03) |
| Aug. 20 | -0.25*** | (0.02) | 0.38 | (0.27) | 1.76*** | (0.09) | 2.46* | (1.03) | 2.51*** | (0.08) | 3.33** | (1.03) |
| Sept. 20 | -0.40*** | (0.02) | 0.43 | (0.27) | 1.57*** | (0.09) | 2.19* | (1.03) | 2.32*** | (0.08) | 3.08** | (1.03) |
| Oct. 20 | -0.39*** | (0.02) | 0.29 | (0.27) | 1.63*** | (0.09) | 2.08* | (1.03) | 2.37*** | (0.08) | 3.00** | (1.03) |
| Nov. 20 | -0.37*** | (0.02) | 0.39 | (0.27) | 1.80*** | (0.09) | 2.42* | (1.03) | 2.49*** | (0.08) | 3.26** | (1.03) |
| Dec. 20 | -0.51*** | (0.02) | 0.27 | (0.27) | 1.94*** | (0.09) | 2.42* | (1.03) | 2.62*** | (0.08) | 3.18** | (1.03) |
| Jan 21 | -0.67*** | (0.02) | -0.01 | (0.27) | 1.90*** | (0.09) | 2.49* | (1.03) | 2.57*** | (0.08) | 3.23** | (1.03) |
| Feb 21 | -0.67*** | (0.02) | -0.10 | (0.27) | 1.58*** | (0.09) | 2.43* | (1.03) | 2.25*** | (0.08) | 3.05** | (1.03) |
| Mar. 21 | -0.61*** | (0.02) | -0.03 | (0.27) | 1.12*** | (0.09) | 1.75+ | (1.03) | 1.65*** | (0.08) | 2.38* | (1.03) |
| N | 4,657,667 |  | 30,611 |  | 4,546,153 |  | 29,328 |  | 4,657,667 |  | 30,611 |  |
| r2p | .478 |  | .447 |  | .405 |  | .355 |  | .455 |  | .406 |  |

Notes: Standard errors in parentheses. + p<0.1, * p<0.05, ** p<0.01, *** p<0.00. ^a^Comorbidities. ^b^Medical offices density, ^c^Hospital beds density ,^d^Municipal marginalization, ^e^ Percentage of urban localities in a municipality, ^f^ January 2020 reference category. r2p=pseudo R-squared. Coefficients are log-odds.
