# Supplementary material for: Socioeconomic Inequalities and Ethnic Discrimination in COVID-19 Outcomes: the Case of Mexico
Source: J Racial Ethn Health Disparities. 2023 Apr 11;11(2):900–12. doi: 10.1007/s40615-023-01571-z (PMC10089566; doi:10.1007/s40615-023-01571-z)
Supplement: Supplementary file 4 — Supplementary file4 (DOCX 17 KB) [file 40615_2023_1571_MOESM4_ESM.docx]

**Table A3** Results from the aggregate OB decomposition stratified by health provider

For those that received care in hospitals managed by the Ministry of Health

|  | **Hosp.** | **%** | **E. Deaths** | **%** | **Deaths** | **%** |
| --- | --- | --- | --- | --- | --- | --- |
| Non-Indigenous | 0.065*** |  | 0.010*** |  | 0.022*** |  |
|  | (0.00) |  | (0.00) |  | (0.00) |  |
| Indigenous | 0.194*** |  | 0.038*** |  | 0.074*** |  |
|  | (0.00) |  | (0.00) |  | (0.00) |  |
| Mean Difference | -0.129*** |  | -0.028*** |  | -0.052*** |  |
|  | (0.00) |  | (0.00) |  | (0.00) |  |
| Explained | -0.109*** | 84.358*** | -0.024*** | 83.423*** | -0.044*** | 85.486*** |
|  | (0.00) | (1.10) | (0.00) | (1.78) | (0.00) | (1.59) |
| Unexplained | -0.020*** | 15.642*** | -0.005*** | 16.577*** | -0.008*** | 14.514*** |
|  | (0.00) | (1.10) | (0.00) | (1.78) | (0.00) | (1.59) |
| N | 3,063,470 | 3,063,470 | 3,032,347 | 3,032,347 | 3,063,488 | 3,063,488 |

For those that received care in hospitals managed by Social Security institutions

|  | **Hosp.** | **%** | **E. Deaths** | **%** | **Deaths** | **%** |
| --- | --- | --- | --- | --- | --- | --- |
| Non-Indigenous | 0.240*** |  | 0.048*** |  | 0.104*** |  |
|  | (0.00) |  | (0.00) |  | (0.00) |  |
| Indigenous | 0.416*** |  | 0.090*** |  | 0.178*** |  |
|  | (0.00) |  | (0.00) |  | (0.00) |  |
| Mean Difference | -0.176*** |  | -0.042*** |  | -0.075*** |  |
|  | (0.01) |  | (0.00) |  | (0.00) |  |
| Explained | -0.140*** | 79.649*** | -0.036*** | 85.679*** | -0.065*** | 86.683*** |
|  | (0.00) | (2.28) | (0.00) | (6.81) | (0.00) | (4.17) |
| Unexplained | -0.036*** | 20.351*** | -0.006+ | 14.321* | -0.010** | 13.317** |
|  | (0.00) | (2.28) | (0.00) | (6.81) | (0.00) | (4.17) |
| N | 1,624,790 | 1,624,790 | 1,543,126 | 1,543,126 | 1,624,782 | 1,624,782 |

Notes: Bootstrapped standard errors in parenthesis (500 replications). Models fitted using an ANOVA-type normalisation and weights from a first-order Taylor linearisation. % Share of each component to the overall gap. + p<0.1, * p<0.05, ** p<0.01, *** p<0.001

## 
