# Supplementary material for: Socioeconomic Inequalities and Ethnic Discrimination in COVID-19 Outcomes: the Case of Mexico
Source: J Racial Ethn Health Disparities. 2023 Apr 11;11(2):900–12. doi: 10.1007/s40615-023-01571-z (PMC10089566; doi:10.1007/s40615-023-01571-z)
Supplement: Supplementary file 5 — Supplementary file5 (DOCX 28 KB) [file 40615_2023_1571_MOESM5_ESM.docx]

**Table A4** Results from the detailed OB decomposition stratified by health provider

For those that received care in hospitals managed by the Ministry of Health

|  | **Hosp.** | **SE** | **E. Deaths** | **SE** | **Deaths** | **SE** |
| --- | --- | --- | --- | --- | --- | --- |
| *Explained component* | | | | | | |
| Demographics | -0.000 | (0.00) | -0.003*** | (0.00) | -0.008*** | (0.00) |
| % | 0.347 | (0.51) | 11.880*** | (1.57) | 16.315*** | (1.12) |
| Comorbidities | -0.048*** | (0.00) | -0.011*** | (0.00) | -0.022*** | (0.00) |
| % | 37.623*** | (0.86) | 39.397*** | (2.74) | 42.348*** | (2.98) |
| NCDs | -0.004*** | (0.00) | -0.001** | (0.00) | -0.001*** | (0.00) |
| % | 3.117*** | (0.35) | 1.774** | (0.60) | 2.289*** | (0.34) |
| Risky Behav. | -0.001 | (0.00) | -0.001+ | (0.00) | -0.001+ | (0.00) |
| % | 0.543 | (0.51) | 2.309 | (1.47) | 1.860+ | (1.01) |
| Med. Attent. | -0.001*** | (0.00) | -0.000* | (0.00) | -0.000** | (0.00) |
| % | 0.392*** | (0.10) | 0.452* | (0.20) | 0.810*** | (0.25) |
| Health Infra. | 0.003+ | (0.00) | -0.003** | (0.00) | -0.004* | (0.00) |
| % | -2.653+ | (1.39) | 11.820** | (3.74) | 8.312* | (3.58) |
| Marginalisation | -0.034*** | (0.00) | -0.000 | (0.00) | 0.001 | (0.00) |
| % | 26.178*** | (1.12) | 1.073 | (5.51) | -2.506 | (4.72) |
| Pop. Disp. | -0.022*** | (0.00) | -0.004*** | (0.00) | -0.007*** | (0.00) |
| % | 16.775*** | (1.74) | 12.681*** | (3.37) | 12.866*** | (3.24) |
| Temporality | -0.003*** | (0.00) | -0.001 | (0.00) | -0.002 | (0.00) |
| % | 2.036*** | (0.49) | 2.036 | (3.63) | 3.192 | (2.52) |
| *Unexplained component* | | | | | | |
| Demographics | 0.001 | (0.00) | 0.000 | (0.00) | 0.000 | (0.00) |
| % | -0.406 | (1.88) | -0.445 | (2.87) | -0.919 | (0.99) |
| Comorbidities | -0.018*** | (0.01) | -0.002+ | (0.00) | -0.004** | (0.00) |
| % | 14.263*** | (3.99) | 7.633+ | (4.36) | 7.504** | (2.72) |
| NCDs | 0.002 | (0.00) | -0.000 | (0.00) | -0.000 | (0.00) |
| % | -1.290 | (2.18) | 0.827 | (2.56) | 0.840 | (1.61) |
| Risky Behav. | 0.001 | (0.00) | -0.000 | (0.00) | -0.000 | (0.00) |
| % | -0.691 | (1.67) | 1.220 | (1.57) | 0.242 | (1.12) |
| Med. Attent. | -0.001 | (0.00) | -0.000 | (0.00) | -0.000 | (0.00) |
| % | 0.838 | (0.77) | 0.617 | (0.65) | 0.340 | (0.59) |
| Health Infra. | -0.006*** | (0.00) | 0.001+ | (0.00) | 0.001 | (0.00) |
| % | 4.897*** | (0.94) | -1.808* | (0.91) | -1.144 | (0.96) |
| Marginalisation | -0.075*** | (0.00) | -0.010** | (0.00) | -0.016*** | (0.00) |
| % | 58.002*** | (3.94) | 36.148** | (11.77) | 31.269*** | (7.06) |
| Pop. Disp. | 0.006*** | (0.00) | 0.000 | (0.00) | 0.000 | (0.00) |
| % | -4.576*** | (1.13) | -0.855 | (0.88) | -0.808 | (0.80) |
| Temporality | 0.194*** | (0.04) | 0.017 | (0.23) | 0.046 | (0.43) |
| % | -150.212*** | (32.26) | -61.335 | (813.88) | -89.243 | (838.26) |
| Intercept | -0.122*** | (0.03) | -0.010 | (0.23) | -0.034 | (0.43) |
| % | 94.817*** | (26.98) | 34.576 | (811.37) | 66.433 | (836.75) |
| N | 3,063,470 |  | 3,032,347 |  | 3,063,488 |  |

For those that received care in hospitals managed by Social Security institutions

|  | **Hosp.** | **SE** | **E. Deaths** | **SE** | **Deaths** | **SE** |
| --- | --- | --- | --- | --- | --- | --- |
| *Explained component* | | | | | | |
| Demographics | -0.043*** | (0.00) | -0.020*** | (0.00) | -0.038*** | (0.00) |
| % | 24.520*** | (1.53) | 48.500*** | (2.25) | 51.558*** | (2.68) |
| Comorbidities | -0.052*** | (0.00) | -0.011*** | (0.00) | -0.021*** | (0.00) |
| % | 29.733*** | (2.01) | 26.090*** | (3.49) | 28.534*** | (2.45) |
| NCDs | -0.008*** | (0.00) | -0.002*** | (0.00) | -0.002* | (0.00) |
| % | 4.279*** | (0.43) | 4.470*** | (1.19) | 2.029+ | (1.06) |
| Risky Behav. | -0.001 | (0.00) | -0.001+ | (0.00) | -0.001*** | (0.00) |
| % | 0.634 | (0.43) | 1.257+ | (0.66) | 1.409** | (0.43) |
| Med. Attent. | 0.000 | (0.00) | -0.000 | (0.00) | -0.000 | (0.00) |
| % | -0.085 | (0.30) | 0.180 | (0.49) | 0.346 | (0.71) |
| Health Infra. | -0.005* | (0.00) | -0.003* | (0.00) | -0.003+ | (0.00) |
| % | 2.956* | (1.37) | 8.070* | (3.32) | 3.633 | (2.25) |
| Marginalisation | -0.021*** | (0.00) | 0.005** | (0.00) | 0.009* | (0.00) |
| % | 11.991*** | (2.28) | -11.572** | (4.19) | -12.047** | (4.62) |
| Pop. Disp. | 0.006*** | (0.00) | 0.001 | (0.00) | 0.002* | (0.00) |
| % | -3.571*** | (0.66) | -1.713 | (2.84) | -2.977* | (1.25) |
| Temporality | -0.016*** | (0.00) | -0.004*** | (0.00) | -0.011*** | (0.00) |
| % | 9.192*** | (1.09) | 10.396*** | (2.23) | 14.199*** | (3.55) |
| *Unexplained component* | | | | | | |
| Demographics | 0.046*** | (0.01) | 0.009** | (0.00) | 0.017*** | (0.00) |
| % | -26.110*** | (6.19) | -20.528* | (8.27) | -22.124*** | (4.16) |
| Comorbidities | -0.046+ | (0.03) | 0.000 | (0.01) | -0.004 | (0.01) |
| % | 26.126+ | (15.22) | -0.023 | (18.29) | 5.893 | (17.50) |
| NCDs | -0.012 | (0.01) | 0.000 | (0.00) | -0.001 | (0.01) |
| % | 6.633 | (5.49) | -0.129 | (5.17) | 1.516 | (7.11) |
| Risky Behav. | -0.012+ | (0.01) | 0.003+ | (0.00) | 0.003 | (0.00) |
| % | 6.601+ | (3.79) | -6.717+ | (3.53) | -4.446 | (4.52) |
| Med. Attent. | 0.009* | (0.00) | 0.002* | (0.00) | 0.003 | (0.00) |
| % | -5.186* | (2.37) | -4.909* | (2.04) | -4.661 | (2.98) |
| Health Infra. | 0.003 | (0.00) | 0.002 | (0.00) | -0.000 | (0.00) |
| % | -1.773 | (2.60) | -4.595 | (3.82) | 0.068 | (4.29) |
| Marginalisation | 0.486*** | (0.05) | -0.008 | (0.01) | 0.037 | (0.02) |
| % | -276.534*** | (31.72) | 18.130 | (30.06) | -48.993 | (32.98) |
| Pop. Disp. | -0.010*** | (0.00) | -0.001 | (0.00) | -0.002 | (0.00) |
| % | 5.803*** | (1.12) | 1.682 | (3.61) | 2.476+ | (1.45) |
| Temporality | -0.047 | (0.27) | 1.647* | (0.79) | 2.638** | (0.86) |
| % | 26.569 | (155.84) | -3899.381* | (1660.82) | -3535.513*** | (1042.82) |
| Intercept | -0.454 | (0.29) | -1.660* | (0.79) | -2.700** | (0.86) |
| % | 258.220 | (170.12) | 3930.790* | (1654.83) | 3619.101*** | (1042.66) |
| N | 1,624,790 |  | 1,543,126 |  | 1,624,782 |  |

Notes: Bootstrapped standard errors in parenthesis (1,000 replications). Models fitted using an ANOVA-type normalisation and weights from a first-order Taylor linearisation. % Share of each component to the overall gap. + p<0.1, * p<0.05, ** p<0.01, *** p<0.001
