# Supplementary figures and images for: Socioeconomic Inequalities and Ethnic Discrimination in COVID-19 Outcomes: the Case of Mexico
Source: J Racial Ethn Health Disparities. 2023 Apr 11;11(2):900–12. doi: 10.1007/s40615-023-01571-z (PMC10089566; doi:10.1007/s40615-023-01571-z)

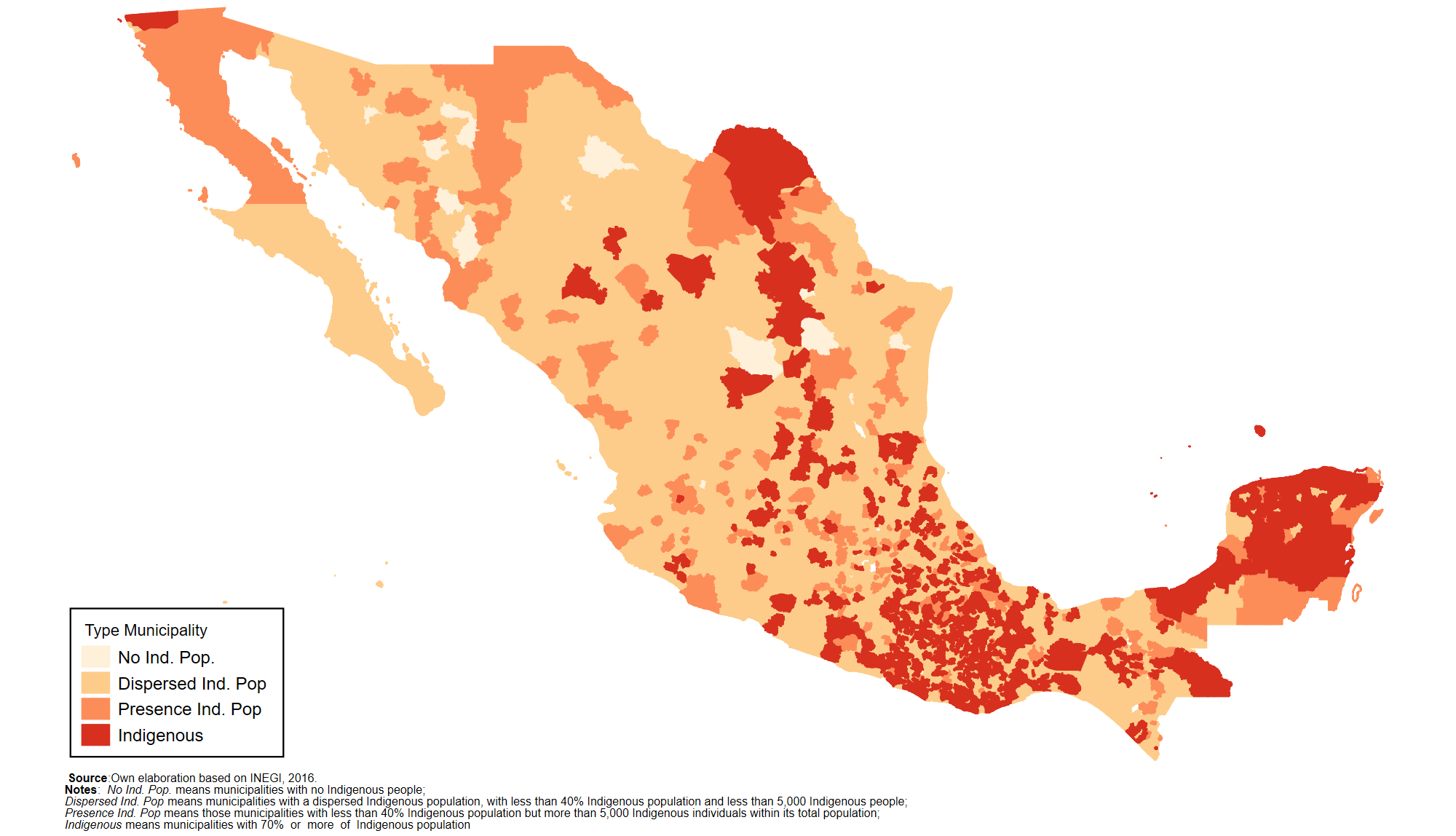

Supplement: Supplementary file 6 — Supplementary file6 (PNG 247 KB) [file 40615_2023_1571_MOESM6_ESM.png]

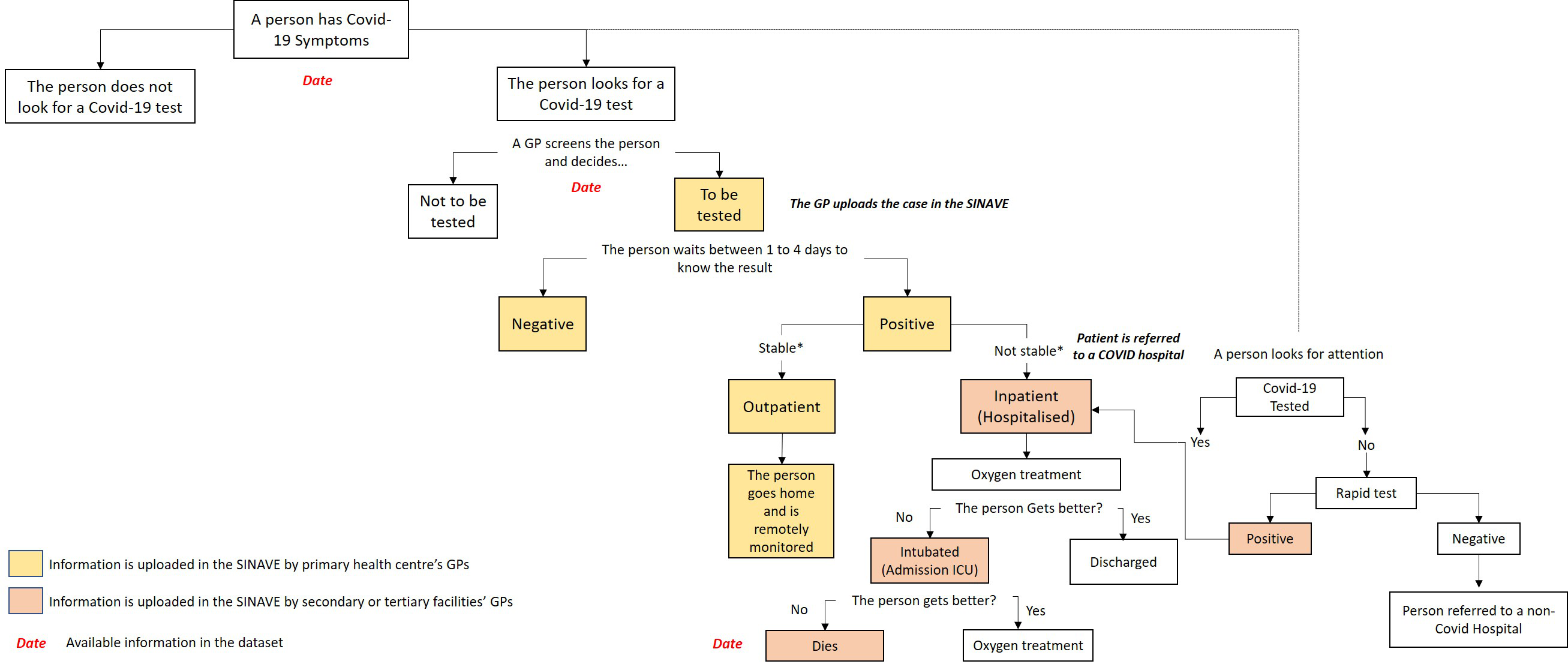

Supplement: Supplementary file 7 — Supplementary file7 (PNG 610 KB) [file 40615_2023_1571_MOESM7_ESM.png]
